# Circadian disruption and cancer- and treatment-related symptoms

**DOI:** 10.3389/fonc.2022.1009064

**Published:** 2022-10-28

**Authors:** Ali Amidi, Lisa M. Wu

**Affiliations:** ^1^ Unit for Psycho-Oncology and Health Psychology, Department of Psychology and Behavioural Sciences, Aarhus University, Aarhus, Denmark; ^2^ Sleep and Circadian Psychology Research Group, Department of Psychology and Behavioural Sciences, Aarhus University, Aarhus, Denmark; ^3^ Aarhus Institute of Advanced Studies, Aarhus University, Aarhus, Denmark; ^4^ Department of Medical Social Sciences, Northwestern University Feinberg School of Medicine, Chicago, IL, United States

**Keywords:** circadian rhythms, cancer, sleep, fatigue, cognitive impairment, depressed mood

## Abstract

Cancer patients experience a number of co-occurring side- and late-effects due to cancer and its treatment including fatigue, sleep difficulties, depressive symptoms, and cognitive impairment. These symptoms can impair quality of life and may persist long after treatment completion. Furthermore, they may exacerbate each other’s intensity and development over time. The co-occurrence and interdependent nature of these symptoms suggests a possible shared underlying mechanism. Thus far, hypothesized mechanisms that have been purported to underlie these symptoms include disruptions to the immune and endocrine systems. Recently circadian rhythm disruption has emerged as a related pathophysiological mechanism underlying cancer- and cancer-treatment related symptoms. Circadian rhythms are endogenous biobehavioral cycles lasting approximately 24 hours in humans and generated by the circadian master clock – the hypothalamic suprachiasmatic nucleus. The suprachiasmatic nucleus orchestrates rhythmicity in a wide range of bodily functions including hormone levels, body temperature, immune response, and rest-activity behaviors. In this review, we describe four common approaches to the measurement of circadian rhythms, highlight key research findings on the presence of circadian disruption in cancer patients, and provide a review of the literature on associations between circadian rhythm disruption and cancer- and treatment-related symptoms. Implications for future research and interventions will be discussed.

## 1 Introduction

Cancer patients suffer from a range of co-occurring side- and late-effects associated with cancer and/or its treatment including fatigue (1), sleep difficulties (2), depressive symptoms (3, 4) and cognitive impairment (5). These cancer- and treatment-related symptoms (CTRS), sometimes described as the “cancer symptom cluster” (6), have a range of negative implications for patients such as delaying cancer treatments, impacting treatment adherence, and detrimental effects on quality of life (7) and daily life functioning (8). Symptoms can be present prior to treatment (9–13), may often worsen during treatment (9, 14–16), and for a large subset, may persist well beyond treatment completion (4, 7, 17–21). Furthermore, CTRS may exacerbate each other’s intensity and development over time (22). The co-occurrence and interdependent nature of these symptoms suggests a possible shared underlying mechanism (23, 24), and while the importance of investigating these symptoms together has been emphasized (25), most research has had a single-symptom focus. Hence, mechanisms underlying CTRS remain unclear.

To date, the predominant hypothesis of a shared underlying mechanism for CTRS has been based on an immune system response (24, 26, 27) as presented in the “sickness behavior model” (6, 28). Sickness behaviors are physiological and behavioral changes, such as fatigue, disturbed sleep and mood, and impaired cognition (6, 27–29) that occur in reaction to an immune response and the release of proinflammatory cytokines such as tumor necrosis factor–α (TNF-α), interleukin (IL)-6 and IL-1β (30). It is generally accepted that inflammation plays an important role in tumorigenesis and that tumor development leads to an intrinsic inflammatory immune response (31). Evidence also suggests that cancer is associated with both immunostimulation and immunosuppression with increased concentrations of various cytokines including TNF-α and IL-6 (32). During the course of treatment, a strong additional inflammatory response may be triggered by both local and systemic therapies such as surgery, radiotherapy and chemotherapies (31, 33, 34). Cancer and treatment-induced immune responses and the release of peripheral proinflammatory cytokines may induce central inflammation mediated by microglial activation within the brain, which can lead to behavioral and cognitive deficits (35). While a meta-analysis supports the sickness behavior model, the strength of association between markers of inflammatory responses and the CTRS varies (30). Furthermore, this model does not generally account for why and how CTRS may persist well beyond the disease and treatment completion, nor does it readily translate into targeted interventions.

Another proposed mechanism of CTRS relates to disruption of the endocrine system and most notably that of the hypothalamic-pituitary-adrenal (HPA) axis. Heightened and chronic stress associated with the cancer disease and its treatment may impact the HPA axis resulting in altered cortisol secretion patterns, which have been associated with CTRS (12, 36, 37). In particular, studies have shown diurnal variations to be altered with evidence of associations between flatter diurnal cortisol slope and more severe CTRS (36, 38, 39). While these lines of evidence underscore the importance of HPA dysregulation as an underlying mechanism of CTRS, these findings may also be closely linked with dysfunction of another fundamental system – the circadian system. Diurnal variations in cortisol reciprocally interact with circadian mechanisms within the brain (40), and thus, disrupted diurnal variations in cortisol may reflect underlying disruptions to this biological timing system.

## 2 Circadian disruption in cancer survivors

Recently, circadian rhythm disruption has emerged as an important and related pathophysiological mechanism underlying CTRS (41–44). Circadian rhythms are endogenous biobehavioral cycles lasting slightly longer than 24 hours in humans and generated by the circadian master clock (45) – the hypothalamic suprachiasmatic nucleus (SCN) (46). The SCN orchestrates rhythmicity in a wide range of bodily functions including rest-activity behaviors, body temperature, immune response, and hormone levels (46, 47). The unique role of circadian rhythms in CTRS is perhaps best demonstrated in animal models in which disturbance of the master clock has resulted in sleep disturbance (48–50), altered mood-related behaviors (51–53) and cognitive impairment (54, 55). In cancer patients, several lines of evidence also support the possible role of circadian disruption in the development of CTRS as will be highlighted in further detail below.

A major appeal of a circadian disruption hypothesis of CTRS is that the expression and regulation of the previously proposed mechanisms of CTRS are reciprocally related to the circadian system. For example, research points to a bidirectional link between circadian rhythms and inflammatory processes (56, 57). On the one hand, the inflammatory immune response may be caused by disrupted circadian rhythms (58). Higher circulating levels of proinflammatory cytokines have been observed in cancer patients with disrupted activity rhythms (59). On the other hand, circadian disruption may occur due to the impact of cytokines on the SCN. Animal studies have shown that proinflammatory cytokines can produce phase shifts in activity rhythms (60), and that TNF-α has a suppressing effect on clock genes with detrimental effects on the circadian system (61). More than a decade ago, there was a call for studies to examine inflammatory responses and circadian rhythms in relation to CTRS to clarify associations and identify points of therapeutic intervention (27).

A bidirectional link between the endocrine and the circadian system is also supported by research. Various endocrine factors are shown to be under direct circadian control (62), including hormones produced by the HPA axis (40, 63), and there’s accumulating evidence to show that chronic disruption of the circadian system may lead to disorders of metabolic, reproductive and mood systems (64). Emerging evidence also suggests that endocrine feedback may play a role in the entrainment of the circadian system. In this regard, altered endocrine functioning has been implicated in the disruption of circadian rhythms likely mediated by altered glucocorticoids and metabolic hormones (65).

Behavioral and psychological alterations following cancer diagnosis and treatment may also independently impact the circadian system either directly through behavioral changes, such as reduced exposure to light (66) or indirectly through the aforementioned pathophysiological mechanisms. There are also well-known bidirectional links between sleep and the immune system (67) with evidence suggesting that both disrupted sleep and long sleep duration is associated with increased systemic inflammation (68). Other psychosocial factors, including stress, anxiety, and depression, are also known to have bidirectional associations with the immune system (69, 70).

**Figure 1:**
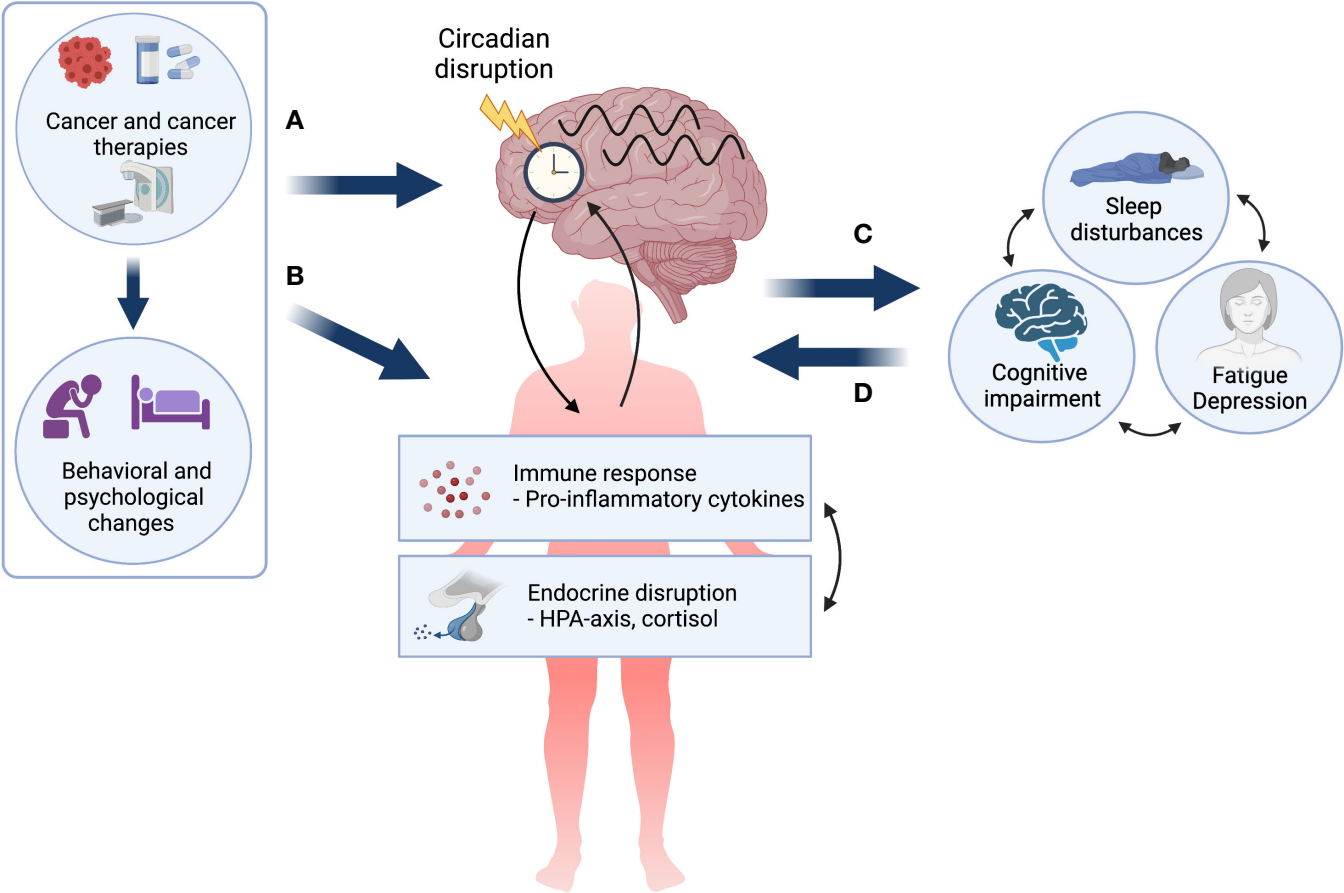
The circadian disruption hypothesis of cancer- and cancer treatment-related symptoms. Cancer and its treatment, as well as associated behavioral and psychological changes may **(A)** directly impact the circadian system resulting in circadian disruption in both biological and behavioral rhythms, and **(B)** lead to a dysregulated immune response and endocrine disruption, which are themselves bidirectionally linked and may both impact the circadian system. Circadian disruption may result in cancer- and treatment-related symptoms (CTRS) or exacerbate pre-existing symptoms **(C)**. Finally, it is important to note that once manifested, chronic CTRS burden may further alter both behavioral and pathophysiological factors creating a self-perpetuating negative loop **(D)**. Created with BioRender.com.

Taken together, a circadian disruption hypothesis of CTRS is not only compatible with other predominant pathophysiological models but adds to them by highlighting the potentially key modulatory role of the circadian system in the manifestation of CTRS (see **Figure 1**). Furthermore, the appeal of the circadian system as an underlying mechanism lies in its modifiability, as it can be targeted in both pharmacological (e.g., melatonin administration) (71) and non-pharmacological interventions (e.g. light therapy) (72, 73) with the potential to stabilize multiple biobehavioral systems and ultimately lead to symptom reduction and improved quality of life.

In the present review, we aim to highlight key research findings of the presence of circadian disruption in cancer patients and provide a detailed review of associations between circadian rhythm disruption and CTRS. Methods of assessment related to CTRS, including patient-reported outcome measures, as well as behavioral and performance-based approaches will be briefly described below. Furthermore, assessment of circadian rhythms through measurement of secretion patterns of melatonin and cortisol, rest-wake activity, and 24-hour body temperature, will be described. Finally, implications for future research and potential interventions to strengthen the circadian system will be discussed.

## 3 Cancer- and treatment-related symptoms

In this section, each of the CTRS will be described and the main methodologies discussed.

### 3.1 Fatigue

Fatigue is among the most prevalent symptoms of cancer and cancer treatment and refers to a “distressing, persistent, subjective sense of physical, emotional, and/or cognitive tiredness related to cancer treatment that is not proportional to recent activity and interferes with usual functioning” (74). It is estimated that between 70 – 90% of cancer patients undergoing radio- or chemotherapy will experience fatigue, and although the number decreases over time, long-term fatigue is prevalent in approximately 30% (75).

The vast majority of studies measuring cancer-related fatigue use patient-reported outcome measures. Although research has identified several biomarkers of fatigue including immune, metabolic, and neuroendocrine markers (76), fatigue is inherently subjective and, thus, most appropriately captured by self-reported measures. Measures of fatigue can be either one- or multi-dimensional. An example of a one-dimensional measure is the widely used Functional Assessment of Chronic Illness Therapy – Fatigue (FACIT-F) (77). An example of an often-used multi-dimensional measure of fatigue is the Multidimensional Fatigue Symptom Inventory (MFSI) (78), which distinguishes between general, emotional, physical, and mental fatigue, as well as vigor.

### 3.2 Sleep problems

Sleep problems are also highly prevalent both during and years after cancer treatments with estimates ranging from 30-50% (79). A variety of methods exist for the assessment of sleep outcomes spanning from patient-reported to actigraphy-based to EEG-defined sleep with polysomnography (PSG). Although the latter method is considered the gold standard to measure objective sleep, PSG is both costly and time-consuming, and therefore less frequently applied in CTRS research.

Because insomnia is subjectively defined, patient-reported measures of sleep quality and insomnia severity have been extensively used in the literature with established cut-offs for determining clinical levels of sleep disturbances. The most widely used measures of patient-reported insomnia severity and sleep quality are the Insomnia Severity index (ISI) and the Pittsburgh Sleep Quality Index (PSQI), which have both been shown to be valid and reliable measures in cancer populations (80, 81).

Another patient-reported measure of sleep behavior can be collected through sleep diaries that require patients to fill out details about the timing and duration of various sleep-related behaviors such as time spent trying to fall asleep, early- and night-time awakenings, and overall time spent in bed. Diaries allow for the extraction of common sleep metrics including sleep onset latency (SOL), wake after sleep onset (WASO), early awakenings (EA), time in bed (TIB), total sleep time (TST), and sleep efficiency (SE).

Actigraphy is yet another measure of sleep behavior often used in cancer populations as it is relatively cost-effective and easy to use, allowing for continuous measurement across longer time periods (82, 83). While actigraphy does not allow for the direct measurement of sleep, rest-activity patterns are good indicators of the timing and duration of sleep (84) and allow for the calculation of common sleep metrics such as SE, WASO and TST. Sleep diaries are often concomitantly collected with actigraphy to edit the rest-activity data.

### 3.3 Depression symptoms

Both during and after cancer treatment, many patients suffer from high psychological distress including symptoms of depression, which may last for years (85). Depending on the method of assessment, prevalence rates across cancer types have been reported to range between 8 – 24% (86). While individual clinical interviews are considered the gold standard for diagnosing depression, due to time- and resource limits, symptoms of depression are most commonly assessed by using validated and reliable self-report scales. Examples of these include the Hospital Anxiety and Depression Scale (HADS) (87) and the Center for Epidemiologic Studies Depression Scale (CES-D) (88), but many more exist (89).

### 3.4 Cognitive impairment

Cognitive impairment refers to changes in mental functions and abilities such as memory decline, and impaired attention and executive functioning. Impairments to cognition are highly prevalent and distressing, and often associated with treatments such as chemotherapy and antihormonal treatment (90), as well as with the cancer disease itself (91), although the underlying mechanisms are still poorly understood.

A neuropsychological test battery is considered the “gold standard” measure of domain-specific cognitive functions. The test battery consists of a range of different standardized and performance-based cognitive tests to assess a patient’s strengths and cognitive weaknesses. Guidelines have been published by the International Cancer & Cognition Task Force with recommended tests to be used in the field of cancer (92).

Although neuropsychological tests are considered to be robust measures of cognitive function, their use is often limited as they are time-consuming and their proper administration requires specialized training. Therefore, self-report measures of cognitive functions are widely used in the research literature using various instruments. A review from 2018 reported considerable diversity in cognitive measures used and found that the two items from the European Organisation for Research and Treatment of Cancer QLQ-C30 (EORTC QLQ-C30) were the most often used items (93). Other common measures included the Functional Assessment of Cancer Therapy-Cognitive Function (FACT-Cog) (94) and the Cognitive Failures Questionnaire (CFQ) (95).

One major limitation of self-report measures of cognitive function is that they are often poor correlates of performance-based neuropsychological tests (96) and instead tend to be more indicative of psychological distress (93). In order to strengthen the scientific rigor of the use of self-report measures of cognitive function, recent recommendations of their use have also been published (97).

## 4 Assessment of circadian rhythms in cancer patients

Circadian rhythm research in cancer patients has typically focused on the measurement of four key markers of circadian rhythms: melatonin, cortisol, activity, and body temperature. Their measurement is described in detail below.

### 4.1 Measurement of melatonin rhythms

Melatonin (5-methoxy-*N*-acetyltryptamine) is a circadian hormone synthesized in the corpus pineale and regulated by the SCN in response to light information received directly through the retinohypothalamic tract (98, 99). As a result of direct anatomical connections between the SCN and the pineal gland, the circadian rhythm of melatonin is considered the best peripheral estimator of the timing of the internal circadian pacemaker (100). In normally entrained individuals, melatonin secretion has a clear circadian rhythm characterized by low levels secreted during the day and a peak in the early morning. Levels typically rise between 8 p.m. and 11 p.m. reaching acrophase between 2 a.m. and 4 a.m. and returning to baseline levels between 8 a.m. and 10 a.m. (101).

The measurement of melatonin concentrations can be undertaken in plasma, serum, urine and/or saliva. For the assessment of circadian phase, plasma is considered the method of choice due to higher values compared with saliva (101). In order to accurately capture the circadian rhythm, it is important to collect samples at regular intervals (e.g., every hour) during the 24-hour day. High frequency blood sampling, thus, requires indwelling canulla in a hospital or laboratory setting. Saliva sampling, on the other hand, is non-invasive and can be undertaken at home, but the drawback is that patients need to be awake during normal sleeping hours for night samples. Alternatively, routine urine sampling in 2 to 8 hour intervals can be used for the measurement of the major metabolite of melatonin, 6-sulphatoxymelatonine. However, given the longer sampling intervals, this method is less accurate when measuring the circadian phase of melatonin secretion (101, 102). The dim light melatonin onset (DLMO) protocol is widely used to assess the melatonin phase. DLMO requires repeated melatonin assessment usually from saliva samples taken every 30 to 60 minutes during evening hours to capture the phase of the evening rise. Although melatonin levels in saliva are generally stable, enabling individuals to store samples at home until delivery to a laboratory, rather strict conditions for collection of samples need to be adhered to that can affect sample quality. For example, while research suggests that 1 hour sampling may be as accurate as 30 minute sampling schemes (103), it is important to initiate sampling several hours before the expected rise. In addition, saliva collection typically needs to occur under dim light conditions or wearing blue light blocking glasses in order to avoid photic melatonin suppression. Individuals also need to avoid food and water 10-15 minutes before sampling times (102, 104, 105), and certain foods, products and drugs ideally ought to be avoided during, at minimum, the sampling period, due to interactions with melatonin levels (including caffeine, alcohol, bananas, chocolate, toothpaste, beta-blockers and non-steroidal anti-inflammatory drugs) (106–111).

### 4.2 Measurement of cortisol rhythms

Cortisol is a glucocorticoid circadian hormone regulated by the HPA axis (112). Cortisol rhythms tend to be diurnal with levels rising early in the morning, then decreasing over the course of the day (113).

The measurement of circadian rhythms in cortisol can be obtained by frequent 24 hour blood serum and plasma sampling (114, 115). However, given the invasive nature of this sampling method, salivary cortisol is the most common method of measuring the amount of unbound, biologically active cortisol in the blood. Most studies use repeated daytime measurements to assess diurnal cortisol rhythms, and thus, possible HPA dysregulation (38). Depending on the variable of interest, different sampling schemes have been recommended. Most commonly used variables include the cortisol awakening response (CAR) (116), diurnal slope (117) and area under the curve (AUC) (118). Irrespective of the variable of interest, it is recommended to collect daily samples on two consecutive days at each time point to increase reliability. For the measurement of CAR specifically, a minimum of three morning samples has been recommended with the first sample being collected at personal awakening time and then 30 and 45 minutes later (119). For diurnal cortisol rhythms, there are unfortunately, as yet, no published consensus guidelines, but the literature recommends the collection of three to six samples across the day for diurnal variables including AUC (117).

### 4.3 Measurement of activity rhythms

In cancer patients, circadian rhythms have mainly been investigated through examination of rest-wake activity rhythms (120). The analysis of inactivity/activity is translated into rest/wake and is based on the observation that there is less movement during rest (or sleep) periods and more movement during wake periods. The rhythm of locomotor activity across the 24 hour day has been described as the circadian activity rhythm (121).

Rest-wake activity is typically measured using an actigraph, a device similar in size to a watch and worn on the wrist. It provides a convenient way to approximate rest versus wake states continuously for 24-hours a day for days, weeks, or even longer (82). A number of circadian parameters can be derived from rest-wake spans including mesor, amplitude, acrophase, rhythm quotient, circadian quotient, peak activity, *R-*squared, *F*-statistics, circadian quotient, interdaily stability, intradaily variability, 24-h autocorrelation (r24), and a dichotomy index (I<O, which is the percentage of activity in-bed that is less than the median activity out-of-bed) (44, 120, 122). See section 4.5 for further details.

### 4.4 Measurement of body temperature rhythms

Core body temperature is another robust marker of the circadian system (123). Core body temperature in homeothermic organisms is regulated around a narrow temperature range with its own distinct rhythm and with an amplitude plateauing between 2 p.m. and 8 p.m. and a minimum temperature in the early morning (124, 125). While the core body temperature rhythm is tightly controlled by the SCN and plays an important role in the coordination of peripheral clocks, the SCN itself has been shown to be resistant to temperature entrainment (126). Research has also shown that the sleep-wake cycle is closely associated with circadian body temperature rhythms (127). In healthy individuals, the sleep period usually occurs when the core temperature curve is decreasing and ends with the rising phase of the curve.

It has been argued that there is no gold standard for the measurement of core body temperature (128). Nevertheless, core body temperature has traditionally been measured in a variety of different sites such as the rectum, the mouth, and the tympanic membrane (128). Continuous measurement of temperature in these sites requires patients to be awake, making it less optimal for 24-hour rhythm assessments. Recently, the development of wireless data loggers has facilitated noninvasive and continuous assessment of both proximal and distal skin temperature without the active involvement of participants (129). While proximal skin temperature (e.g. forehead, thigh, stomach) is positively correlated with core body temperature, distal skin temperature (e.g. hands, feet) is inversely associated with core body temperature (130). It is also known that distal skin temperature is phase advanced with respect to core body temperature (129), suggesting that heat loss from extremities may drive the circadian rhythm of core body temperature.

### 4.5 Methodological considerations for the analysis of circadian markers

Depending on the methods of assessment and sampling rate frequencies, various methods and statistical approaches exist for analyzing rhythmic data to determine important rhythm parameters and circadian rhythm disruption. While it is beyond the scope of the present paper to review all approaches, a few key approaches will be highlighted here.

Both parametric and non-parametric approaches have been developed to analyze circadian rhythm markers (131). An example of the former is cosinor analyses, which use the method of least squares to fit a cosine curve to periodic 24 hour data. Common metrics derived from this method to analyze markers of circadian rhythms (e.g. melatonin, cortisol, rest-activity, and temperature) include the *mesor*, the rhythm-adjusted mean; the *amplitude*, the difference between the peak and the wave mean; the *period*, the duration of one cycle; and the *acrophase*, the time of day of peak activity. Another variable sometimes reported and that represents overall circadian rhythm robustness is the pseudo F-statistic, which is based on the residuals from cosine fitting models (132, 133).

A limitation of the above methods to assess circadian rhythms, however, is that there are no established cut-offs or thresholds to readily determine circadian disruption. Thus, circadian disruption is often operationalized by employing general linear models to assess between-group differences or changes over time in these measures (134, 135). Furthermore, the application of cosinor-based methods may be better suited to some circadian markers than others. Although commonly used with actigraphy-based rest-activity assessments, motor activity patterns, for example, do not typically resemble a sinusoid, and thus, other approaches have been warranted (131, 136).

To overcome some of these challenges, non-parametric approaches to circadian activity rhythms have been developed with the aim to assess intra-daily variability as a marker of sleep-wake cycle disturbances, and inter-daily stability as a marker of circadian entrainment (131). One promising approach in cancer populations has been the use of the dichotomy index (I<O). The I<O is a measure of the relative amount of activity in-bed below the median of activity out-of-bed (137). Lower I<O is considered to reflect weaker rest-activity rhythmicity (138) and studies have shown that lower I<O is associated with poorer outcomes in cancer patients (136, 139). A strength of the I<O is the reporting of general cut-off values. An I<O value close to 100% is indicative of non-disrupted rest-activity rhythms as seen in healthy subjects, whereas a median value of 97.5% has been reported in cancer patients and considered the threshold for circadian rest-activity disruption (140). Finally, more sophisticated non-parametric approaches have also been applied to rest activity data, such as Hidden Markov Modelling that can i) threshold activity into different states in a probabilistic way and in a time dependent manner, ii) capture square wave forms observed in activity data alongside heterogeneous ultradian variances in human activity, and iii) can generate circadian rhythm parameter estimates based on probabilities of transitions between rest and activity (141).

Finally, given that circadian markers are often measured continuously across time, dynamical modelling that describe the state of the rhythm as a function of time capturing the ongoing fluctuations or change in the rhythms may also be applied, although in practice these approaches are less widely used (142).

## 5 Circadian rhythm disruption in cancer patients: Key research findings

In the following section, key research findings related to the assessment of each of the circadian markers in cancer populations will be presented and associations with CTRS will be reviewed.

### 5.1 Melatonin levels in cancer patients

Disrupted melatonin rhythms have been observed in a wide variety of diseases (143–146). Unfortunately, research regarding the effects of cancer and cancer treatments on circadian melatonin rhythms have been sparse, possibly due to the aforementioned methodological challenges associated with assessing melatonin rhythms. However, there are notable and relatively consistent patterns of findings from the few, small studies that exist. A recent study that compared salivary melatonin levels in newly diagnosed prostate cancer patients with controls found that the cancer patients had lower melatonin levels compared with the controls (147). Breast cancer patients have also been found to excrete lower levels of melatonin from 24-hour urine samples (148) and have exhibited suppressed nocturnal peak, mesor, and amplitude of serum melatonin when compared with benign patient groups (149). Melatonin rhythms and secretion levels have also been examined over the course of cancer treatment. Among early-stage breast and ovarian cancer patients receiving chemotherapy, studies have found significant reductions in the level of night-time melatonin over the course of chemotherapy (150, 151). Melatonin has also been examined in other cancer types including cervical cancer (152), lung cancer (153, 154), and colorectal cancer (155). Typically, these studies have found lower melatonin concentrations than patient or healthy control groups, though two studies found differences from healthy controls in circadian melatonin profiles as well, including a flatter slope (152). Using a DLMO protocol, a small recent study found indications for earlier melatonin secretion in gastrointestinal cancer patients with disrupted activity rhythms (140). However, it ought to be mentioned that inter-subject variability was markedly larger for cancer patients than controls, and such variability highlights a potential weakness of the DLMO protocol.

#### 5.1.1 Melatonin and CTRS

Few studies have specifically investigated the association between circadian melatonin rhythms and CTRS (see **Table 1**). Chang and colleagues (154) investigated diurnal variation in salivary melatonin in newly-diagnosed lung cancer patients prior to treatment compared with matched healthy controls. Although lung cancer patients evidenced lower melatonin levels and flatter diurnal slopes than controls, there were no significant associations observed between melatonin slope or melatonin levels and sleep quality, symptoms of depression, or fatigue. In another study (156), serum melatonin levels were investigated in a group of newly diagnosed breast cancer patients. Pre-surgical levels were negatively associated with self-reported symptoms of depression, while melatonin levels post-surgery were negatively associated with daytime sleepiness. Clearly, more research is needed with the aim of prospectively investigating associations between the development of CTRS and melatonin rhythms. Although there are evident methodological challenges in capturing circadian melatonin rhythms, the DLMO protocol may be useful for capturing the slope of dim-light melatonin secretion and phase shifts in cancer-patients throughout the cancer treatment trajectory (102).

**Table 1:** Summary of studies that examined melatonin and cancer- and treatment-related symptoms (CTRS).

Authors	Fatigue	Sleep	Depressed mood	Cognition	Patient population	Stage of cancer trajectory/assessment times	Melatonin markers	Outcome measures	Study design	Found association between melatonin and CTRS	Results
Chang and Lin (154)	X	X	X		Lung cancer (n=40) and healthy controls (n=31)	Newly diagnosed	Salivary melatonin 3 times/day: diurnal melatonin slope and levels	Fatigue: BFI Sleep: PSQI Depression: HADS	cross-sectional	no	Although cancer patients had lower melatonin levels and flatter slopes than controls, there were no associations between melatonin slope or levels with fatigue, sleep or depression scores.
Li et al. (151)		X			Breast cancer (n=180)	T1: Stage I-III awaiting chemotherapy T2: During first cycle T3: At last cycle of chemotherapy	Urinary aMT6s levels	Sleep: PSQI, actigraphically-assessed nighttime sleep duration, sleep efficiency, nighttime total wake time	longitudinal	not tested	Sleep efficiency significantly lower than at baseline, but higher than beginning of chemotherapy. Deterioration in morning urinary aMT6s level during chemotherapy was cumulative. Did not examine associations between CTRS and melatonin levels.
Zaki et al. (156)		X	X		Breast cancer (n=45)	T1: Newly diagnosed, before surgery T2: Daytime post- surgery T3: Nighttime post-surgery	Serum melatonin levels	Sleep: ISI, ESS Depression: BDI	cross-sectional	yes	Pre-surgery melatonin levels negatively correlated with depression scores. Daytime post-surgery melatonin levels negatively correlated with daytime sleepiness.

aMT6s, 6-sulfatoxymelatonin; BDI, Beck Depression Inventory; BFI, Brief Fatigue Inventory; HADS, Hospital Anxiety and Depression Scale; ISI, Insomnia Severity Index; ESS, Epworth Sleepiness Scale; PSQI, Pittsburgh Sleep Quality Index.

### 5.2 Cortisol levels in cancer patients

In a broad array of studies focused predominantly on breast cancer and ovarian cancer patients, increased disruption to cortisol rhythms or secretion levels has been found based on comparisons with control groups or patients at an earlier stage of disease. The predominant finding is that compared with comparison groups, the primary cancer groups tend to experience elevations in mean or nocturnal cortisol levels (38, 157–159) and flatter diurnal cortisol rhythms (38, 157, 159). A study that followed ovarian cancer patients prior to primary treatment to 1 year post-treatment, found that patients showed significant reductions in nocturnal salivary cortisol secretion and plasma IL-6 and a more normalized diurnal cortisol rhythm at 6 months with changes maintained at 1 year (160). In studies of lung cancer patients, similar findings of loss of circadian rhythmicity have been found when compared with healthy controls (161, 162).

#### 5.2.1 Cortisol and CTRS

Research focused on cortisol and CTRS has primarily focused on salivary cortisol (as opposed to urinary, serum or plasma cortisol) and examined diurnal cortisol slope, cortisol awakening response or cortisol levels at a particular point in time (e.g., morning or nocturnal levels) (see **Table 2**). Numerous studies have examined associations between markers of cortisol rhythms and depressed mood in cancer patients at different stages of the cancer trajectory, primarily among breast cancer patients, but also among lung, colorectal, gynecologic, and prostate cancer patients (38, 59, 154, 160, 164–168, 170–172, 175, 177, 179, 180). The findings have been equivocal with many studies finding no association, including among newly diagnosed lung, endometrial and breast cancer patients (154, 175, 179), advanced breast cancer patients (115), and breast cancer survivors (164, 166, 180). Others *have* found associations, including associations between evening cortisol levels in ovarian cancer patients and depressive symptoms both before and after primary treatment (38, 160, 172), higher morning cortisol levels in women with metastatic breast cancer (177), and reduced diurnal variation in cortisol levels among depressed advanced metastatic cancer inpatients compared with those who were non-depressed (170). The cortisol awakening response has also been found to be blunted in depressed metastatic breast cancer patients compared with those who were non-depressed (165). In contrast, a study by Kuhlman (171) found the opposite; the cortisol awakening response positively predicted changes in depressed mood over time in early stage breast cancer patients. Sephton also found, contrary to expectations, that accentuated diurnal cortisol rhythms were associated with greater depressed mood (177).

**Table 2:** Summary of studies that examined cortisol and cancer- and treatment-related symptoms (CTRS).

Authors	Fatigue	Sleep	Depressed mood	Cognition	Patient population	Stage of cancer trajectory/assessment times	Cortisol markers	Outcome measures	Study design	Found association between cortisol and CRTS	Results
Abercrombie et al. (159)				X	Breast cancer (n=17) and healthy controls (n=31)	metatastic	3-day salivary cortisol, 4 times/day: diurnal cortisol slope, mean cortisol levels	Cognition: RAVLT	cross-sectional	no	No associations between cortisol slope and cognitive functioning among patients.
Alexander et al. (163)	X	X	X		Breast cancer (n=200)	after primary treatment	24-h urinary cortisol	Fatigue: FACT-F, BFS, FCS, SCID for CRF Depression: HADS	cross-sectional	yes	No differences in urinary cortisol between fatigued and non-fatigued patients.
Bower et al. (36)	X				Breast cancer, fatigued (n=13) and non-fatigued (n=16)	1 to 5 years after diagnosis	2-day salivary cortisol, 4 times/day: diurnal cortisol slope, mean cortisol levels, AUC	Fatigue: RAND SF-36 – energy/fatigue subscale	cross-sectional	yes	Fatigued survivors had significantly flatter cortisol slope than non-fatigued survivors, less rapid decline in in evening hours. Survivors with highest fatigue had flattest cortisol slopes.
Castonguay et al. (164)	X		X		Breast cancer (n=145)	T1: ≤20 weeks after primary treatment T2: 3 months later T3: 6 months later T4: 9 months later T5: 12 months later	3 non-consecutive days (T1) or 2 nonconsecutive days (T2-T5) salivary cortisol: AUC	Fatigue: BFI Depression: CES-D	longitudinal	no	Fatigue did not significantly predict intra-individual changes in physical activity or cortisol. Depressive symptoms significantly predicted physical activity but not cortisol levels.
Chang and Lin (154)	X	X	X		Lung cancer (n=40) and healthy controls (n=40)	Newly diagnosed	Salivary cortisol 3 times/day: diurnal cortisol slope and levels	Fatigue: BFI Sleep: PSQI Depression: HADS	cross-sectional	yes	Cortisol slope and fatigue were significant predictors of PSQI score. Flatter cortisol slope (and higher fatigue) predicted more severe sleep disturbance. Anxiety and depression were not influenced by cortisol rhythms.
Cuneo et al. (39)	X				Ovarian cancer (n=30)	At least 5 years post-diagnosis	3-day salivary cortisol, 3 times/day: diurnal cortisol slope	Fatigue: POMS-SF fatigue subscale	cross-sectional	yes	Flatter diurnal cortisol slopes were associated with significantly higher fatigue after controlling for age and cancer stage.
Giese-Davis et al. (165)			X		Breast cancer, nondepressed (n=45) and depressed (n=45)	Metastatic T1: assessed 1 week before T2: day of, and T3: day after Trier Social Stress Test	2-day salivary cortisol, 5 times/day: baseline diurnal cortisol slope, mean waking cortisol, mean wake + 30 rise	Depression: PANAS	longitudinal + manipulation	yes	Compared with nondepressed patients, depressed patients had lower 2-day average baseline waking rise in log cortisol level. No other differences in cortisol between groups at any time.
Ho et al. (166)		X	X		Breast cancer (n=181)	Non-metastatic or no recurrence	2-day salivary cortisol, 4 times/day	Sleep: 10-point scale of sleep quality, time of awakening, total sleep hours	cross-sectional	yes	Controlling for initial cortisol level, flatter diurnal cortisol slope associated with later time of awakening, poorer sleep quality, shorter total sleep hours. Depression and anxiety not correlated with slope.
Hoyt et al. (167)		X	X		Prostate cancer (n=66)	T1: Localized and treated in prior 2 years T2: 4 months later	3-day salivary cortisol, 4 times/day: diurnal cortisol slope, AUC and CAR	Sleep: PSQI Depression: CES-D	longitudinal	yes	Mediation models examining sleep at T1 on depression at T2 with BMI and age as covariates: Flatter cortisol slope and less overall cortisol output related to higher depressive symptoms. No indirect effect for CAR. Reverse mediation models - i.e., cortisol –> sleep quality –> depression was non-significant or small indirect effects
Hsiao et al. (168)		X	X		Breast cancer (n=62)	T1: Aged 40 and below who completed treatment T2: 2^nd^ month T3: 5^th^ month T4: 8^th^ month	1-day salivary cortisol, 6 times/day: diurnal cortisol slope	Sleep: MOS sleep scale Depression: BDI-II	longitudinal	mixed	Diurnal cortisol slopes were steeper compared with baseline. Significant decreases in depression, but no significant changes in sleep variables across the 8 months. Habitually later time of awakening over the 8 months predicted flatter cortisol slope. Habitual time of going to bed, sleep problem index, and depression not associated with cortisol patterns during 8 month follow up.
Huang et al. (169)	X		X		Hepatocellular cancer (n=75) and healthy controls (n=33)	Outpatients not under surgical treatment	3-day salivary cortisol, 5 times/day: mean daily cortisol, mean cortisol levels at each sampling time point, peak cortisol levels, cortisol slope, AUC	Sleep: PSQI	cross-sectional	yes	Patients with higher daily cortisol levels tended to report poorer sleep quality. This was not case with healthy controls. Poor sleepers among patients with least severe liver disease showed slight increase in cortisol level at bedtime. Bedtime cortisol level of poor sleepers with least severe liver disease was higher than that of healthy poor sleepers. (Note: all patients with more severe disease had poor sleep).
Jehn et al. (170)			X		Cancer patients, with (n=31) and without depression (n=83)	Advanced metastatic cancer	Plasma cortisol at 8am and 8pm: cortisol VAR	Depression: HADS-D	cross-sectional	yes	Relative cortisol VAR decreased in depressed patients compared with non-depressed patients. Cortisol VAR independently negatively associated with depression.
Kuhlman et al. (171)			X		Breast cancer (n=135)	T1: Recently diagnosed T2: 6 months after primary treatment	72-h salivary cortisol, 4 times/day: CAR, diurnal cortisol slope, AUC	Depression: CES-D	longitudinal	yes	No cortisol indices were associated with depressive symptoms at T1. After controlling for T1 depression, CAR predicted depressive symptoms at 6 months. When history of major depression was included as covariate, association between CAR and increases in depressive symptoms trended in the same direction but were not significant.
Lutgendorf et al. (172)			X		Ovarian cancer (n=112) and tumors of low malignant potential (n=25)	Awaiting surgery	3-d salivary cortisol, 4 times/day: CAR, diurnal cortisol slope, AUC	Depression: CES-D	cross-sectional	yes	Elevations in total depression and vegetative depression were related to higher evening cortisol.
Mormont et al. (173)	X	X	X		Colorectal cancer (n=200)	Metastatic before chronotherapy	2-day serum cortisol at 8am and 4pm: amplitude	Fatigue: EORTC QLQ-C30 v2. Depression: HADS	cross-sectional	not tested	Examined associations between cortisol circadian rhythms and rest-activity rhythms only. Cortisol circadian rhythms were positively correlated with r24 but not I<O or mean activity.
Palesh et al. (174)		X			Breast cancer (n=99)	Metastatic	2-day salivary cortisol, 5 times/day: cortisol slope, AUC	Sleep: actigraph-assessed time in bed, SOL, SE, nocturnal wake episodes, WASO	cross-sectional	yes	Longer nocturnal wake episodes associated with flatter diurnal cortisol slope. No significant relationships between 2-day mean of waking cortisol or cortisol rise and other measures of sleep.
Rich et al. (59)	X		X		Colorectal cancer, normal (high r24, n=40) and dampened 24-h rest-activity patterns (low r24, n=40)	Metastatic before chronotherapy	2-day serum cortisol at 8am and 4pm: amplitude	Fatigue: EORTC QLQ-C30 v2. Depression: HADS	cross-sectional	not tested	Examined associations between r24 status and cortisol only. High r24 patients had higher serum cortisol ratios between 8am and 4pm than low r24 patients.
Sannes et al. (175)			X		Endometrial cancer (n=82)	Nonmetastatic, before surgery	3-day salivary cortisol, 4 times/day: diurnal cortisol slope, intradindividual variability	Depression: SIGH-AD	cross-sectional	yes	Depressive symptoms unrelated to cortisol slope. After controlling for presence of poorer prognosis cancer subtypes, greater depressive symptoms were significantly related to greater cortisol intraindividual variability.
Schmidt et al. (176)	X				Breast cancer (n=265)	T0: pre-adjuvant treatment T1: week 7 T2: post-intervention week 13	1-day salivary cortisol, 5 times/day: CAR, AUC, diurnal cortisol slope	Fatigue: FAQ	longitudinal	yes	**Cross-sectional results:** Higher evening cortisol levels associated with higher physical fatigue levels T0 and T1. Larger AUC associated with higher physical fatigue levels. At T0, physical fatigue associated with flatter cortisol slope and higher CAR, but not at T1 and T2. **Longitudinal results:** Significant positive associations of change in evening cortisol level and AUC with change in physical fatigue, but no association with change in morning cortisol, CAR, or slope. Changes in affective or cognitive fatigue not associated with changes in cortisol parameters.
Schrepf et al. (160)	X		X		Ovarian cancer (n=117)	T1: Prior to surgery T2: 6 months T3: 1 year	3-day salivary cortisol, 3 times/day: mean cortisol, diurnal cortisol slope	Fatigue: POMS-SF Sleep: how many hours of sleep? Depression: CES-D (vegetative symptoms)	longitudinal	yes	At 6 months, reductions in nocturnal cortisol secretion and more normalized diurnal cortisol rhythm, maintained at 1 year. Reductions in nocturnal cortisol associated with declines in fatigue, and marginally with vegetative depression.
Sephton et al. (177)			X		Breast cancer (n=72)	Metastatic	3-day salivary cortisol, 4 times/day: diurnal mean cortisol level, diurnal cortisol slope	Depression: CES-D	cross-sectional	yes	Depression scores were uncorrelated with mean cortisol levels. Patients with greater depression had higher morning cortisol and accentuated diurnal cortisol rhythms.
Tell et al. (178)	X	X	X		Breast cancer (n=130)	Recently diagnosed, after surgery	2-day salivary cortisol, 5 times/day: wake-up cortisol, CAR, linear and quadratic slope from wake-up to bedtime	Fatigue: MFSI-SF Sleep: PSQI	cross-sectional	yes	Significant associations between ongoing fatigue and sleep quality and cortisol parameters: Women reporting greater ongoing fatigue had higher cortisol levels upon awakening Elevation in ongoing fatigue associated with a less pronounced CAR, slower decline over the day. Poor sleep quality associated with linear slope (flatter), but not associated with cortisol upon awakening, CAR or quadratic change. Reduction in sleep quality predicted slower cortisol decline in linear slope.
Vedhara et al. (179)			X		Breast cancer (n=85) and healthy controls (n=59)	Newly diagnosed	2-day salivary cortisol, 4 times/day: AUC, diurnal cortisol, early morning peak	Depression: HADS	cross-sectional	yes	No associations between cortisol variables and depressed mood or distress among cancer patients.
Weinrib et al. (38)	X		X		Ovarian cancer (n=100), benign disease (n=77), healthy women (n=33)	Suspected ovarian cancer	3-day salivary cortisol, 3 times/day: diurnal cortisol variability, nocturnal cortisol	Fatigue: POMS fatigue subscale Depression: CES-D	cross-sectional	yes	Ovarian cancer patients had significantly elevated nocturnal cortisol and diminished cortisol variability compared with women with benign disease and healthy women. Among cancer patients, higher nocturnal cortisol and less cortisol variability associated with greater functional disability, fatigue, and vegetative depression.
Zeitzer et al. (115)		X			Breast cancer (n=97) and healthy controls (n=24)	Advanced	28-h plasma cortisol at 20-60 minute intervals: Diurnal variation, phase, amplitude, mesor, phase angles	Sleep: polysomnography	cross-sectional	yes	The circadian pattern of cortisol (timing, timing relative to sleep, or amplitude) was indistinguishable between patients and controls. There was an aberrant spike of cortisol during the sleep of a subset of women, during which there was an eightfold increase in the amount of objectively measured wake time. This cortisol aberration was associated with shorter disease-free interval.

AUC, Area Under the Curve; BDI, Beck Depression Inventory; BFI, Brief Fatigue Inventory; BFS, Bidimensional Fatigue Scale; CAR, Cortisol Awakening Response; CES-D, Center for Epidemiologic Studies Depression Scale; cortisol VAR, relative diurnal variation of cortisol; EORTC QLQ-C30 v.2, The European Organization for Research and Treatment of Cancer Core Quality of Life Questionnaire version 2; FACT-F, Functional Assessment of Cancer Therapy: Fatigue; FAQ, Fatigue Assessment Questionnaire; FCS, Fatigue Catastrophising Scale; HADS, Hospital Anxiety and Depression Scale; HADS-D, Hospital Anxiety and Depression Scale – Depression; MFSI-SF, Multidimensional Fatigue Symptom Inventory – Short Form; MOS, Medical Outcomes Study; PANAS, The Positive and Negative Affect Schedule; POMS, Profile Of Mood States; PSQI, Pittsburgh Sleep Quality Index; r24, autocorrelation coefficient at 24 hours; RAVLT, Rey Auditory Verbal Learning Test; SCID for CRF, Structured Clinical Interview for the Diagnostic and Statistical Manual – IV to diagnose Cancer-Related Fatigue; SE, sleep efficiency; SF-36, 36-item Short-Form Survey; SIGH-AD, Structured Interview Guide for the Hamilton Depression Inventory; SOL, sleep onset latency; WASO, wake after sleep onset.

More consistent associations between markers of cortisol rhythms and fatigue and sleep quality have been found (36, 38, 39, 115, 154, 160, 166, 174, 176, 178). Flatter diurnal cortisol slopes have been associated with greater fatigue in breast cancer patients post-surgery (178), pre-adjuvant treatment (176), and 1 to 5 years after diagnosis (36) and in ovarian cancer survivors (39), as well as poorer sleep quality in breast cancer survivors (166, 174, 178) and among newly diagnosed lung cancer patients (154). Higher cortisol upon awakening has also been associated with fatigue in breast cancer patients evaluated post-surgery (178) and higher daily cortisol levels with poorer sleep quality among hepatocellular cancer patients (169). In a large, longitudinal study of 265 breast cancer patients undergoing adjuvant therapies (176), higher *evening* cortisol levels were associated with higher physical fatigue both pre-adjuvant therapy and 7 weeks later. Importantly, this study evaluated changes in cortisol levels over time and found associations between changes in evening cortisol levels and AUC with changes in physical fatigue from pre-adjuvant therapy to 13 weeks later, though neither morning cortisol, the cortisol awakening response, nor slope were associated with fatigue.

Highlighting the interrelationships between different CTRS, Hoyt (167) found that lower cortisol output and a flatter diurnal slope accounted for 45-57% of the effect of sleep quality at study entry upon depressed mood 4 months later in prostate cancer survivors.

Not all studies have found associations between cortisol rhythms and CTRS. For example, a large study (n=200) of breast cancer patients after primary therapy that measured 24-hour urinary cortisol instead of diurnal salivary cortisol, found no differences between fatigued and non-fatigued patients (163). Abercrombie etal. (159) investigated metastatic breast cancer patients and found no association between cortisol slope and cognition.

### 5.3 Activity rhythms in cancer patients

Circadian activity rhythm disruption has been detected across the cancer trajectory. Soon after diagnosis, many cancer patients undergo surgery. In one study of 60 endometrial cancer patients, significant rest-activity disruption (as measured by lower mesor and weaker amplitude) 1 week and 1 month post-surgery was found, with significant recovery on all parameters by 4 months post-surgery (181). Furthermore, the cancer group had more impaired rhythms than a reference group at 1-week post-surgery suggesting that surgery may also be associated with circadian disruption. A large majority of research in this area has focused on circadian activity rhythm disruption associated with chemotherapy, particularly in breast cancer patients. In one such longitudinal study, circadian impairments were examined in breast cancer patients before and during chemotherapy (182). Ninety-five women scheduled to receive neoadjuvant or adjuvant anthracycline based chemotherapy for stage I-III breast cancer wore wrist actigraphs for 72 consecutive hours pre-chemotherapy, and during weeks 1, 2 and 3 of cycles 1 and 4 of chemotherapy. Compared to baseline, amplitude, mesor, up-mesor, down-mesor, and rhythmicity were all significantly impaired during the first week of both chemotherapy cycles with some recovery during weeks 2 and 3. However, most variables remained significantly more impaired than baseline during weeks 2 and 3 of cycle 4. These findings were corroborated by another longitudinal study that included a cancer-free control group (14). One hundred and forty-eight women with stage I-III breast cancer scheduled to receive at least 4 cycles of chemotherapy and matched cancer-free controls participated. Circadian activity rhythm data was collected *via* 72 consecutive hour actigraphy before the start of chemotherapy, at the end of cycle 4 of chemotherapy, and 1 year after the start of chemotherapy. *R-*squared was the circadian outcome of interest indicating rhythm robustness. At baseline, breast cancer patients had more disrupted rhythms than the controls. At cycle 4, the cancer patients had more disrupted rhythms compared to their own baseline levels and to controls. At 1 year, cancer patients’ circadian activity rhythms did not differ from non-cancer controls. The number of chemotherapy cycles also appear to be important. One study examined rest-activity in newly diagnosed breast cancer patients during chemotherapy cycles (183). Average scores of all rhythm parameters (i.e., mesor, amplitude, acrophase, rhythm quotient, circadian quotient, peak activity, dichotomy index, and autocorrelation coefficient) significantly decreased with an increasing number of chemotherapy cycles. In addition, activity rhythm disruptions during chemotherapy are likely to peak at the start of the cycles and decrease during the periods between cycles (120).

Other studies have found circadian activity rhythm disruptions in other cancer populations or associated with other cancer treatments (including mixed cancer patients undergoing chemotherapy and/or radiation therapy, colorectal cancer patients undergoing chemotherapy, gynecologic cancer patients undergoing chemotherapy, and breast cancer patients undergoing endocrine therapy). Such studies have generally shown disruptions to circadian parameters when compared with pre-treatment, the beginning of treatment, with cancer controls, or with healthy controls (184–189). Studies have also investigated activity rhythms in lung cancer populations at different stages of the cancer trajectory (190–192). In one longitudinal study of 82 newly diagnosed lung cancer patients undergoing cancer treatment (193), sleep-wake rhythms were assessed at baseline prior to treatment and at four subsequent time points at weeks 6, 12, 24, and 48. While poorer sleep-wake rhythms were observed at baseline, significant improvements were observed at week 48.

Even years after cancer treatment, circadian activity rhythm alterations have been detected. One small scale study of breast cancer survivors found circadian activity rhythm alterations 5 years after primary diagnosis when compared with a healthy control group (194).

Overall, numerous studies suggest that circadian activity rhythms may be disrupted prior to, during and after cancer treatment. In addition, a recent scoping review of actigraphy-based circadian activity rhythms revealed that up to 55% of patients with advanced cancer had disrupted activity rhythms (195).

#### 5.3.1 Activity rhythms and CTRS

Numerous studies have elucidated potential associations between important circadian rhythm markers and various CTRS, typically through the use of actigraphy over 24 to 72 hours of continuous measurement (see **Table 3**). Studies on the associations between circadian activity rhythms and CTRS have been undertaken in cancer populations across the cancer trajectory.

**Table 3:** Summary of studies that examined rest-activity and cancer- and treatment-related symptoms.

Authors	Fatigue	Sleep	Depressed mood	Cognition	Patient population	Stage of cancer trajectory	Rest-activity markers	Outcome measures	Study design	Found association between rest/activity and CRTS	Results
Ancoli-Israel et al. (196)	X	X	X		Breast cancer (n=85)	Newly diagnosed stage I-III scheduled to receive chemotherapy	72-h actigraphy: F statistic, acrophase	Fatigue: MFSI-SF Depression: CES-D Sleep: PSQI, FOSQ	cross-sectional	no	No significant correlations between rhythm variables and fatigue, sleep, depression, or functional outcome of sleep.
Ancoli-Israel et al. (14)	X	X	X		Breast cancer (n=68) and cancer-free controls (n=60)	T1: Newly diagnosed stage I-III scheduled to receive chemotherapy T2: end of cycle 4 T3: 1 year post-chemotherapy	72-h actigraphy: R-squared	Fatigue - MFSI-SF; Sleep - total sleep time, total nap time; PSQI global sleep quality score; Depression - CES-D	longitudinal	not tested	T1: Patients longer total daytime nap time, worse sleep quality, more fatigue, more depression, and more disrupted rest-activity rhythms than controls; T2: Patients worse sleep, increased fatigue, more depression, more disrupted rest-activity rhythms compared to T1 and to controls. T3: Patients’ fatigue, depression returned to T1 levels, but still worse than controls; nap time and rest-activity rhythms did not differ from controls.
Ancoli-Israel et al. (197)	X	X	X	X	Breast cancer (n=69) and matched controls (n=64)	T1: Newly diagnosed stage I-III scheduled to receive chemotherapy T2: end of cycle 4 T3: 1 year post-chemotherapy	72-h actigraphy: R-squared	Fatigue: MFSI-SF Depression: CES-D Sleep: PSQI, TST, WASO, %sleep, NAPTIME Cognition: Neuropsychological test battery, PAOFI	longitudinal	yes	No significant group-by-time interaction in objective cognition. Significant group-by-time interaction in self-reported cognition: significant decrements from Baseline to Cycle 4 and 1 year among breast cancer patients but no marked changes in controls. Decreases in objective cognitive functioning in cancer patients predicted by less robust circadian activity rhythms, worsening sleep quality and increases in nap time compared to baseline.
Berger et al. (185)	X	X			Colon and rectal cancer (n=14)	Stage II-III during chemotherapy	7-day actigraphy: mesor, amplitude, acrophase, circadian quotient, r24	Fatigue: Piper Fatigue Scale Sleep: PSQI	cross-sectional	not tested	Disturbed sleep, low daytime activity, and impaired rest-activity rhythms during the first week after chemotherapy cycles 1–3. Rhythm measures were 78%–83% (mesor) and 66%–72% (amplitude) of values obtained in healthy young adults. Rhythm consistency from day to day (r24) was 0.33-0.34 during the first week after chemotherapy 1–3.
Berger et al. (198)	X		X		Breast cancer (n=190) randomized to behavioral therapy sleep intervention or healthy eating control group	T1: Newly diagnosed stage I-IIIA 3-4 weeks before chemotherapy T2: During cycle 3 T3: 30 days after last cycle	7-day actigraphy: mesor, amplitude, peak activity, acrophase, circadian quotient, r24	Fatigue – Piper Fatigue Scale, Daily Fatigue Intensity (Item #7 from Piper) Depression - HADS	longitudinal intervention	yes	Rhythm parameters more disrupted compared to healthy adults. More robust rhythms associated with lower fatigue, depressive symptoms, BMI and higher performance status.
Berger et al. (199)	X	X			Breast cancer (n=130)	Stage I-IIIA during 48 hours before chemotherapy	≤48-h actigraphy: mesor, amplitude, acrophase, goodness of fit	Fatigue: Piper Fatigue scale Sleep: PSQI	cross-sectional	yes	Worse sleep quality and more impaired components of sleep quality, sleep latency, and habitual sleep efficiency correlated with lower mesor. Longer subjective sleep latency was the only component associated with lower amplitude.
Cash et al. (200)			X		Head and neck cancer (n=55)	Newly diagnosed before chemoradiation	6-day actigraphy: r24; I<O (nighttime restfulness); acrophase	Depression: PHQ-9 depressive symptoms	cross-sectional	yes	Cognitive and affective depression symptoms associated with rest-activity rhythm disruption. Overall and somatic depression symptoms associated with phase shifts, shifting from morning to evening. *Rest/activity rhythm disruption and lower nighttime restfulness, but not acrophase, associated with 2-year overall survival.*
Chang and Lin (193)	X	X	X		Lung cancer (n=82)	T1: Newly diagnosed T2: 6 weeks T3: 12 weeks T4: 24 weeks T5: 48 weeks after start of treatment	72-h actigraphy: I<O	Fatigue: BFI Sleep: PSQI Depression: HADS	longitudinal	not tested	Compared with baseline, sleep–wake rhythms improved significantly after starting treatment. Fatigue worsened significantly; depressive symptoms improved.
Chen et al. (192)		X			Lung cancer (n=106)	Post-treatment	72-h actigraphy: r24, I<O	Sleep: Actigraph-assessed TST, SE, SOL	cross-sectional	yes	Significant positive correlations of the TST with I<O, SOL with UP activity mean. Negative correlations of SE with UP activity mean, SOL with I<O, SOL with 24-h light-activity minutes. Lower I<O, lower 24-hour light-activity minutes, and higher value for the UP activity mean had poorer objective sleep quality.
Du-Quiton et al. (201)		X	X		Non-small cell lung cancer inpatients (n=42) and outpatients (n=42)	Advanced stage before or beginning of chemotherapy	3 to 7-day actigraphy during first chemotherapy cycle (inpatients) or prior to chemotherapy (outpatients): mesor, amplitude, acrophase, circadian quotient, rhythm quotient, peak activity, r24, day-night activity balance, night-day sleep balance	Depression: HADS	cross-sectional	yes	Outpatients: - more robust daily activity patterns (mean daily activity, daily amplitude, peak of activity) and longer, more consolidated nighttime sleep (night-day sleep balance) compared with inpatients. - more disrupted daily sleep-activity rhythms associated with worse depression and/or anxiety. - severely depressed outpatients - lower activity levels during daytime and more active during night than those with lower depression scores. - inactivity during the day and daytime napping associated with depression; - higher circadian amplitudes of activity, and higher peak daily activity associated with less depressed mood. - better night-day sleep balance, more nighttime sleep and less daytime sleep were associated with lower depression scores. Inpatients: No associations between circadian rhythms and depression.
Grutsch et al. (191)	X	X		X	Lung cancer (n=84)	Advanced stage in period prior to and during first treatment	4 to 7-day actigraphy: mesor, amplitude, acrophase, circadian rhythm robustness and day-to-day stability, peak activity, r24	Fatigue, Sleep, Cognition: EORTC fatigue, sleep, & cognitive subscales	cross-sectional	yes	Insomnia severity correlates negatively with 24-hour autocorrelation, day-to-day stability. Outpatient fatigue levels associated with diminished robustness of circadian quotient, rhythm quotient, night-day balance of time spent asleep. More robust day-night activity/sleep measurement differences, the less fatigue these patients experience during each day.
Innominato et al. (202)	X	X			Cohort 1: Metastatic colorectal cancer (n=237) Cohort 2: Histologically proven advanced or metastatic cancer requiring medical treatment (n=31)	Metastatic or advanced post-treatment	72-h actigraphy: I<O	Cohort 1 – Fatigue, Sleep: EORTC QLQ-C30 v2 Cohort 2 – Fatigue, Sleep: MDASI	cross-sectional	yes	Cohort #1: Significantly lower I<O associated with greater fatigue and sleep trouble. Greater circadian disruption associated with more severe fatigue and sleep problems. Cohort #2: Significantly lower I<O associated with greater fatigue, but not sleep disturbance. Greater circadian disruption associated with higher fatigue.
Levin et al. (190)	X	X	X		Non-small cell lung cancer (n=33)	Stage IIIA, IIIB-IV before or during chemotherapy	4 to 7-day actigraphy: circadian amplitude; circadian fragmentation/ amplitude of ultradian rhythms; circadian quotient; peak activity; I<O, rhythm quotient	Fatigue: EORTC QLQ- C30 Sleep: PSQI Depression: EORTC QLQ-C30, QLI Cognition: EORTC QLQ-C30	cross-sectional (part of intervention study)	not tested	No correlation between performance scores and any actigraphy data item. Patients reported poor sleep quality as well as fatigue 20-points below population-based surveys.
Li et al. (151)		X			Breast cancer (n=180)	T1: Stage I-III awaiting chemotherapy T2: During first cycle T3: At last cycle of chemotherapy	Percent rhythm, F-statistic, amplitude, mesor, acrophase	Sleep: PSQI, actigraphically-assessed nighttime sleep duration, sleep efficiency, nighttime total wake time	longitudinal	not tested	Sleep efficiency significantly lower than at baseline, but higher than beginning of chemotherapy. Percent rhythm, F-statistic, and mesor at end of chemotherapy were significantly lower than baseline. Did not examine associations between CTRS and rest-wake activity.
Liu et al. (122)	X				Breast cancer (n=148) and healthy controls (n=61)	T1: Stage I-III before chemotherapy T2: After 4 cycles of chemotherapy	72-h actigraphy: amplitude, acrophase, mesor, up-mesor, down-mesor, F-statistic	Fatigue: MFSI-SF	longitudinal	yes	Increases in fatigue significantly associated with greater disruptions in amplitude, mesor, and F-statistic over time.
Ma et al. (203)		X			Cancer (n=68)	Advanced stage, at least 3 months post-surgery	72-h actigraphy: r24, I<O	Sleep: PSQI; 3 d sleep log; actigraphy-assessed TIB, TST, SE, WASO, SOL, waking episodes	cross-sectional	yes	Actigraphy-assessed sleep parameters (TST, SE, SOL, WASO but not TIB, waking episodes) correlated with rhythm parameters (r24 and I<O). Rest/activity rhythms of patients with poor sleep quality (PSQI>5) were much less regular than those with good sleep quality. r24 significantly predicted sleep quality (negative association).
Miaskowski et al. (204)	X	X			Breast, prostate, lung, or brain cancer (n=185)	Before radiation therapy	24-h actigraphy: mesor, amplitude, acrophase, r24	Fatigue: LFS Sleep: PSQI, GSDS	cross-sectional	yes	Significant correlations between PSQI global score and SOL, TST, sleep period time, mesor, and circadian quotient. Significant correlation between fatigue subscores and acrophase.
Mormont and Waterhouse (205)*	X		X		Colorectal cancer (n=200)	Metastatic, before chronotherapy	3 to 5-day actigraphy: mean activity level, r24, I<O	Fatigue: EORTC QLQ-C30 v2. Depression: HADS	cross-sectional	yes	Fatigue was associated with all activity scores. Depression was associated with dampened rhythm parameters (r24 and I<O)
Ortiz-Tudela et al. (188)	X				Cancer (n=49)	T1: Advanced stage, before chronotherapy T2: During chronotherapy T3: Right after chronotherapy T4: Late after chronotherapy	13-day actigraphy spanning the four time points: I<O, r24, interdaily stability, intradaily variability, relative amplitude	Fatigue: NCI CTC-AE v3.0	longitudinal	yes	Circadian disruption (i.e., I<O ≤ 97.5%) during or after chemotherapy associated with significantly higher risk of clinically significant fatigue.
Palesh et al. (206)		X			Colorectal cancer with sleep problems (n=155) and no sleep problems (n=82)	Metastatic, before treatment	72-h actigraphy: I<O, clock time of lowest activity using cosinor analysis, average activity counts	Sleep: TST, SE, sleep latency, WASO	cross-sectional	yes	Patients with sleep complaints had worse circadian function (i.e., lower I<O) compared to those without sleep problems (96.4% vs 98.1%) with clinical cut off of 97.5%.
Rich et al. (59)	X		X		Colorectal cancer, normal (high r24, n=40) and dampened 24-h rest-activity patterns (low r24, n=40)	Metastatic, before chronotherapy	3-day actigraphy: r24 (high = top quartile, low = bottom quartile), I<O, mean activity	Fatigue: EORTC QLQ-C30 v2. Depression: HADS	cross-sectional	yes	High r24 patients had significantly fewer fatigue symptoms than low r24 patients, but no differences in anxiety and depression.
Roscoe et al. (207)	X		X		Breast cancer (n=78) undergoing chemotherapy or radiation or both (but not overlapping) randomized to either paroxetine or placebo	T1: 72 h after second on-study treatment T2: 72 h after fourth on-study treatment	Actigraphy: r24, mean activity	Fatigue - FSCL, MAF Depression: CES-D, HDI, POMS	longitudinal	yes	r24, mean activity significantly correlated with percent sleep and mostly significantly correlated with fatigue, mood and depression. Change scores in r24 and mean activity over time were in general significantly correlated with changes in fatigue, mood, depression (unrelated to paroxetine).
Sultan et al. (183)	X			X	Breast cancer (n=25)	T1: Newly diagnosed during cycle 1 chemotherapy T2: During cycle 2 chemotherapy T3: During cycle 3 chemotherapy	72-h actigraphy: mesor, amplitude, acrophase; rhythm quotient, circadian quotient, peak activity, I<O, r24	Fatigue, Sleep, Cognition: Hindi QLQ-C30	longitudinal	no	Significant decrease on all functional and symptom scales that include fatigue, insomnia and emotional domains from cycle 1 to cycle 6.

*Note that this study overlaps with Mormont et al. (173) so have omitted one from this table.

CES-D, Center for Epidemiologic Studies Depression Scale; EORTC QLQ-C30 v.2, The European Organization for Research and Treatment of Cancer Core Quality of Life Questionnaire version 2; FOSQ, Functional Outcomes of Sleep Questionnaire; FSCL, the Fatigue Symptom Checklist; GSDS, General Sleep Disturbance Scale; HADS, Hospital Anxiety and Depression Scale; HDI, Hamilton Depression Inventory; I<O, actigraphic dichotomy index; LFS, the Lee Fatigue Scale; MAF, the Multidimensional Assessment of Fatigue; MDASI, MD Anderson Symptom Inventory; MFSI-SF, Multidimensional Fatigue Symptom Inventory – Short Form; NCI CTC-AE v3.0, National Cancer Institute Common Terminology Criteria for Adverse Events version 3.0; PAOFI, Patient Assessment of Own Functioning Inventory; PHQ-9, Patient Health Questionnaire-9; POMS, Profile of Mood States; PSQI, Pittsburgh Sleep Quality Index; QLI, Ferrans and Powers Quality of Life Index; r24, autocorrelation coefficient at 24 hours; SE, sleep efficiency; SOL, sleep onset latency; TIB, time in bed; TST, total sleep time; WASO, wake after sleep onset.

Several studies have revealed associations between circadian activity disruption and CTRS prior to treatment onset (115, 196, 199, 200, 204–206). More disrupted circadian activity rhythms have been found to be associated with greater depressed mood prior to treatment among head and neck cancer patients and lung cancer patients (200, 201). A study of metastatic colorectal cancer patients prior to chronotherapy also found that patients with a high r24 coefficient (i.e., greater regularity) had fewer fatigue symptoms than those with a low r24 coefficient (59). In a study of breast cancer patients prior to chemotherapy, lower mesor (i.e, mean level of activity) was associated with worse sleep quality and higher sleep onset latency (199). A study of a mixed group of cancer patients before treatment, also found a limited number of significant correlations between circadian activity rhythm markers and sleep quality (204). However, another study of breast cancer patients scheduled for chemotherapy did not find associations between circadian activity rhythms and CTRS of fatigue, sleep quality or depression (196).

Many studies have examined circadian activity rhythms and CTRS during cancer treatment (14, 122, 183, 185, 188, 190, 193, 198, 201, 208). An early study by Roscoe and colleagues (207) directly examined and found significant temporal associations between increases in circadian activity disruption across cycles of chemotherapy and increases in depression and fatigue among breast cancer patients undergoing chemotherapy. Another study focused on depression, this time in lung cancer patients, found associations between disrupted sleep-activity rhythms and worse depression among outpatients prior to chemotherapy, but not among inpatients during chemotherapy (201). A subsequent study by Liu and colleagues of 148 Stage I-III breast cancer patients undergoing chemotherapy, also found that more disrupted circadian activity rhythms were significantly associated with increases in fatigue (122). Other cross-sectional studies have had similar findings (188, 191).

Disturbances to circadian rhythms have also been associated with CTRS post-treatment. For example, in a cross-sectional study by Chen and colleagues (192) of 106 lung cancer patients, poorer circadian function, including a lower dichotomy index, was associated with poorer objective sleep quality. A recent study examined circadian activity rhythms and cognition in breast cancer patients during and after treatment. There was a significant group-by-time effect in self-reported, but not objective cognition when compared with matched controls. Changes in objective cognitive functioning were positively associated with changes in circadian rhythmicity (i.e., a decrease in cognitive functioning at follow-up was predicted by reduced circadian activity rhythm robustness, worsening sleep quality, and increases in nap time compared to baseline (197).

Finally, among cohorts of advanced cancer patients, significant associations have been detected between disrupted circadian activity rhythms and fatigue (202, 205), depressed mood (205), and poorer subjective sleep/sleep quality (203, 206).

### 5.4 Temperature rhythms in cancer patients

Thus far, research on circadian temperature rhythms in cancer patients has been sparse. In one small observation study of 9 breast cancer survivors (209), circadian core body temperature was measured using an ingested radio telemetry pill. Results were suggestive of circadian disruption of skin temperature in all participants. However, due to the lack of a comparison group, larger controlled studies are indicated. Another small study involving 10 breast cancer patients receiving chemotherapy used wireless skin surface temperature patches on the front thorax (210). Half of the patients exhibited disrupted circadian skin surface temperature rhythms following chemotherapy. In a recent study, significantly deteriorated chest surface temperature rhythms were observed in gastrointestinal cancer patients (N = 25) with disrupted activity rhythms as indicated by a low dichotomy score (< 97.5%) compared with patients without such disruptions (140).

#### 5.4.1 Temperature rhythms and CTRS

To the best of our knowledge, no studies have specifically examined the relationship between circadian temperature rhythms and CTRS.

## 6 Discussion

This review describes key findings of studies that have examined circadian rhythms in cancer patients and associations with CTRS. The majority of studies focused on circadian activity rhythm disruptions in cancer patients and many found associations between activity rhythm disruptions and fatigue, sleep and depressed mood. A number of studies also examined cortisol and CTRS in cancer, particularly by examining diurnal variation or cortisol levels. The findings were more mixed, especially with respect to associations with depressed mood. However, apart from a couple of exceptions, more consistent associations were found between indicators of cortisol disruption (including flatter diurnal cortisol slopes and higher cortisol levels at different times of the day) and fatigue and sleep outcomes. Few studies examined melatonin levels in cancer patients across time, and even fewer examined associations with CTRS, which is surprising given the current interest in exogenous melatonin as a potential antiproliferative agent for some cancers (211). Cognition was rarely examined in any of the reviewed studies, with only one finding associations between circadian activity rhythm disruption and cognitive impairment.

For the most part, the reviewed studies have focused on one or maybe two approaches to the assessment of circadian rhythms. Studies in this area would likely benefit from a multi-modal approach to the assessment of circadian rhythms, e.g., through the use of advanced actigraphy that includes measurement of multiple markers, such as activity and skin temperature rhythms. In addition, longitudinal studies assessing multiple circadian rhythms and associations with CTRS over time would provide richer data regarding the nature and strength of these associations. Furthermore, the inclusion of health or non-cancer control groups would provide the field with a clearer picture of circadian rhythm changes, and associations with side effects and symptoms that are unique to the cancer patient experience. The field would also benefit from further work to develop an operationalized standard for what a normative healthy circadian rhythm ought to be, so that there are clearer cut-offs for determining clinically significant circadian rhythm disruption. In conclusion, given the potential modifiability of the circadian system through enhancement of both photic and non-photic zeitgebers, targeting the circadian system in the treatment of CTRS is a fertile area for future research.

Overall, we have highlighted the important role that the circadian system may play in the manifestation of CTRS. A limitation of this review is that we did not review the potential role of circadian disruption on mortality. Indeed, there have been numerous seminal studies that have found associations between circadian markers and mortality in cancer patients, and that deserve mention due to their obvious relevance to this topic (e.g., 136, 173, 212, 213). Pioneering work by Mormont and colleagues (173) examined circadian rest-activity rhythms in 192 metastatic colorectal cancer patients receiving chronomodulated chemotherapy after failure of a first treatment protocol. Survival at two years was five times higher in patients with stronger activity rhythms (I<O in upper quartile) than those with weaker activity rhythms (I<O in lower quartile). A later study reinforced these findings in 192 previously untreated metastatic colorectal cancer patients undergoing chronomodulated chemotherapy (139). A pooled study of 436 patients that included the aforementioned cohorts plus an additional cohort of colorectal cancer patients, the majority of whom had failed prior chemotherapy for metastatic disease, confirmed that I<O was a robust predictor of overall survival, particularly among those with an I<O above 97.5% (136). Important studies focusing on cortisol markers in cancer patients have also found associations with survival. In studies by Sephton and colleagues (213, 214) that examined 104 metastatic breast cancer patients and 62 lung cancer patients, diurnal cortisol slope positively predicted survival after seven and across three years respectively. However, these findings need to be considered in light of poor correlation between cortisol concentrations in the serum and in saliva, particularly in the case of metastatic colorectal cancer (212). Thus, future studies would benefit from further examination of associations between rigorous markers of circadian rhythms and survival, in addition to CTRS.

A further limitation of this review is that it focused on circadian rhythm disruption at the physiological and behavioral levels. We did not examine disruption at molecular and/or cellular levels. For example, there is research showing that clock gene variations, particularly to NPAS2, CLOCK, RORA, RORB, and PER3, may contribute to small but statistically significantly elevated cancer risk (215). In addition, disrupted cellular signaling pathways in cancer patients (e.g., of the mechanistic target of rapamycin [mTOR]) may be controlled by the circadian clock (216), and thus may also underlie CTRS.

### 6.1 Future directions and conclusion

**Figure 2:**
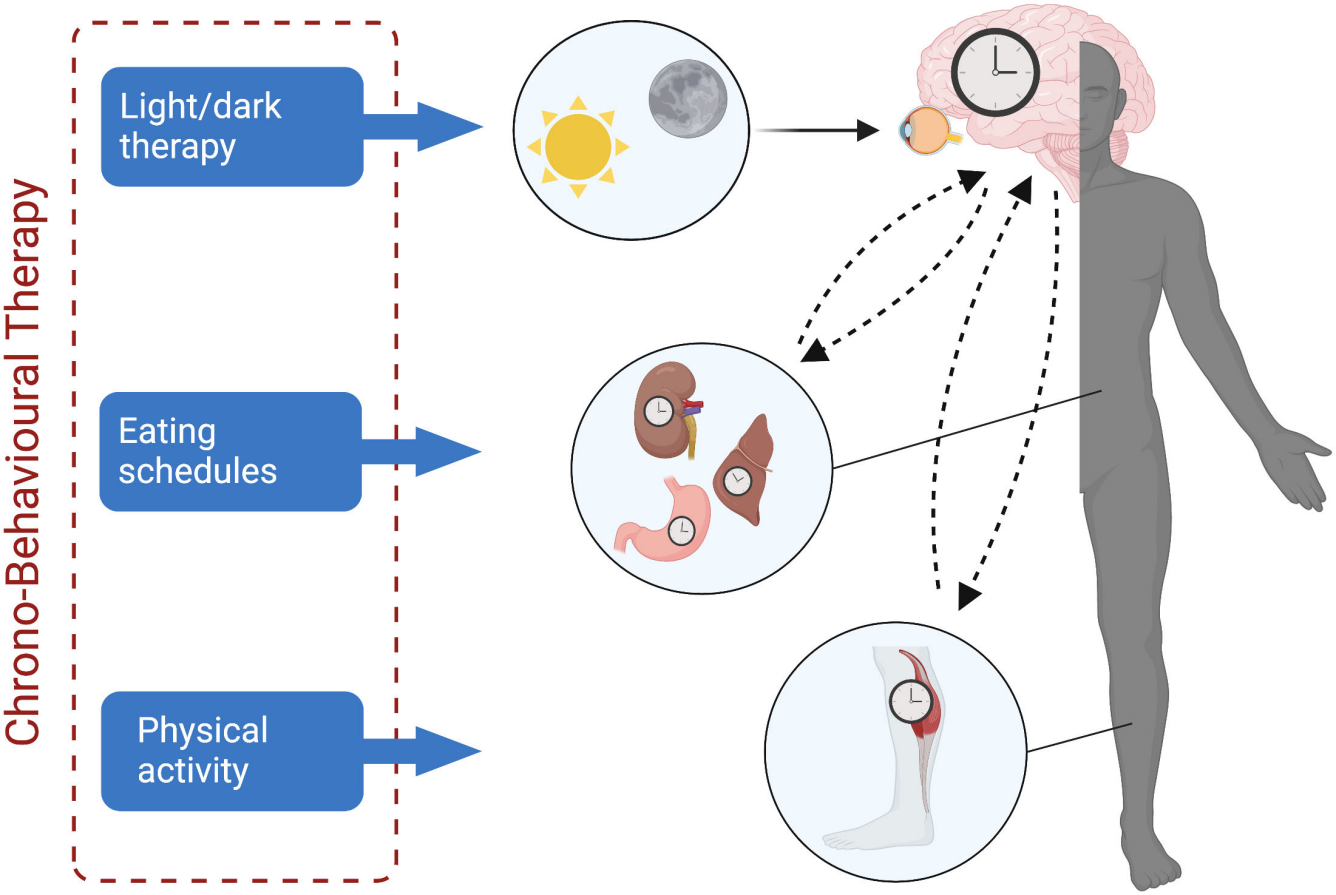
Entrainment of central and peripheral clocks through targeted interventions. Strengthening of the circadian system through direct entrainment of the central clock (i.e., the suprachiasmatic nucleus) may occur through implementation of light/dark therapy; entrainment of peripheral clocks may occur through interventions that target the timing of eating and physical activity. Created with BioRender.com.

Overall, this review suggests that circadian rhythms may be disrupted in cancer patients, and that such disruptions may contribute to the development and persistence of CTRS. In this regard, the circadian system offers a potential modifiable target for a variety of pharmacological and non-pharmacological interventions that aim to normalize circadian rhythms and, thus, ameliorate CTRS. Importantly, synchronization of circadian rhythms to the external environment occurs through entrainment *via* exposure to environmental “zeitgebers” or time-givers. Such zeitgebers include bright light, which potently drives the SCN rhythm, and non-photic zeitgebers (e.g., physical activity, timing of eating), which may drive rhythms of peripheral systems (217–219). Under healthy conditions, the central SCN rhythm directly coordinates peripheral rhythms through endocrine and autonomic nervous system signals and regulation of core body temperature, and indirectly through feedback from activity and feeding rhythms (219). Misalignment occurs if the central rhythm is misaligned to the light/dark cycle or if central and peripheral rhythms are not aligned with each other (220), which can impair the homeostasis of the body (219) and potentially contribute to CTRS. Importantly, the receptivity of circadian rhythms to zeitgebers illustrates how the circadian system is inherently *modifiable*, making it an attractive intervention target. Thus, the enhancement of central and peripheral zeitgebers may be a pathway to improving circadian health in cancer patients and, in turn, CTRS. In this regard, the optimization of the timing of multiple zeitgebers in cancer patients through what we term “*Chrono-Behavioral Therapy*” may be an approach worth investigating in future research (as conceptualized in **Figure 2** below).

Light (both natural and artificial) is the strongest, direct zeitgeber of the SCN (i.e., the central clock of the circadian system), and has been used as a therapeutic tool to treat other disorders, including seasonal affective and other mood disorders for decades already (221). Thus, it is not a surprise that there has been a particular focus on light and its association with CTRS. A study by Liu and colleagues (66) assessed circadian activity rhythms with actigraphy in breast cancer patients who were undergoing chemotherapy. Increased fatigue was significantly associated with decreased light exposure, possibly due to patients spending less time outdoors in bright light. This work triggered a range of intervention studies that tested the use of light exposure to treat CTRS (72, 73, 222–225). In general, protocols instruct cancer patients to use a light box or glasses emitting circadian stimulating light each morning upon waking for 30-45 minutes for 4 weeks or during treatment in order to improve the robustness of the circadian system. Results have shown that light therapy can prevent fatigue and depression in cancer patients undergoing treatment (222, 224), and ameliorate fatigue and improve sleep in cancer survivors after primary treatment (72, 73, 223, 226). Unfortunately, these studies have generally not been sufficiently powered to determine if circadian rhythms mediate light therapy’s effect on CTRS, but one study did determine that bright light therapy protected breast cancer patients from experiencing circadian activity rhythm deterioration during chemotherapy (227).

Another potential area of work focuses on enhancement of peripheral zeitgebers including the timing of physical activity and the timing of eating. Physical activity is a strong non-photic zeitgeber for the mammalian circadian clock (228) likely in part due to effects on central clock genes in skeletal muscles that regulate biological processes (229). Non-photic zeitgebers may support the circadian system through associative learning processes that engage circadian time as a conditioned stimulus (217, 230). In addition, non-photic behavioral zeitgebers tend to be salient to the individual and can serve as a “gatekeeper” to photic zeitgebers (i.e., light/dark exposure) (217). Indeed, there is evidence that physical activity, particularly at night, can phase delay circadian rhythms (i.e., shift the circadian rhythm to later) (231–234). A recent systematic review also confirmed exercise’s phase-shifting properties across studies (235). The timing of eating is another potential peripheral zeitgeber of the circadian system (219, 236) *via* homeostatic effects on core body temperature (237). Importantly, metabolic dysfunction is a comorbidity of many types of cancers and implicated in peripheral fatigue (238). Furthermore, circadian misalignment can occur if food intake occurs during the dark phase, resulting in systemic metabolic dysregulation (219, 239, 240). Both animal and human research indicates that later timing of food intake may result in negative health outcomes (241–243). Indeed, a recent study found that night eating during the COVID-19 pandemic was associated with greater swings in fatigue (244).

In the field of psychiatry, attempts have already been made to harness the power of peripheral zeitgebers through a therapeutic approach called “interpersonal social rhythm therapy,” originally developed to treat patients with bipolar disorder (245). The therapy is based on the hypothesis that bipolar disorder arises due to dysregulated neurotransmitter systems and perturbations in the circadian system, and therefore focuses on behavioral techniques to improve the regularity of a person’s daily routines. Thus far, interpersonal social rhythm therapy has been found to be feasible and satisfactory in patients with bipolar disorder, but has not yet been proven to be efficacious as more rigorous randomized controlled studies are yet to be undertaken (246). It has not yet been evaluated in cancer patients.

A final point to consider is the potential of telemonitoring for the assessment of circadian rhythms and CTRS in the future. For the most part, the measurement approaches described in the studies reviewed in this paper are not used in routine clinical practice, likely due to the difficulties and expense of collecting and tracking patient data in real-time. However, in recent years, the rapid evolution of wearable sensor technology, E-Health applications, and cloud-based computing have made the implementation of new IT-based health care management methods possible (247, 248). Indeed, a number of recent studies have demonstrated the feasibility of telemonitoring of circadian markers (including rest-wake and biological) *and* patient-reported outcomes of cancer patients in their own homes (140, 249–252). Thus, the effect of an increased interest in circadian rhythms and health combined with the wave of popularity of new health monitoring technology, has provided the research and health care community with optimal conditions for telemonitoring research to grow. Furthermore, such work would likely form a solid basis for a precision health approach to cancer patient care into the future.

Thus far, the medical field has already attempted to harness the circadian system in cancer treatment itself through chronotherapy approaches that time drug delivery to the appropriate phase of the circadian rhythm with varying degrees of success (215). Our review adds to that important work by summarizing the increasing body of work linking circadian disruption with CTRS, and thus, it highlights the potential of the circadian system as an important target for clinical monitoring and interventions in the future with the ultimate goal of improving cancer patients’ quality of life.

## Author contributions

AA and LW were responsible for conceptualization of this review and contributed to the scientific analysis of existing research. Both authors contributed equally to manuscript preparation, and read and approved the final submitted version.

## Funding

AA’s effort was supported by grants from the Danish Cancer Society (R174-A11447-17-S52) and Independent Research Fund Denmark (5053-00220B). LW’s effort was supported by the European Union’s Horizon 2020 Research and Innovation Programme under the Marie Sklodowska-Curie grant agreement no. 754513 and the Aarhus University Research Foundation, as well as by the American Cancer Society award number 131642-RSG-18-053-01-PCSM.

## Conflict of interest

The authors declare that the research was conducted in the absence of any commercial or financial relationships that could be construed as a potential conflict of interest.

## Publisher’s note

All claims expressed in this article are solely those of the authors and do not necessarily represent those of their affiliated organizations, or those of the publisher, the editors and the reviewers. Any product that may be evaluated in this article, or claim that may be made by its manufacturer, is not guaranteed or endorsed by the publisher.
